# Molecular and Pharmacokinetic Rationale for the Use of *Chelidonium majus* L. in Wound Healing: An In Silico and In Vitro Validation

**DOI:** 10.3390/molecules31081320

**Published:** 2026-04-17

**Authors:** Ana Borges, Carlos Seiti H. Shiraishi, Rui M. V. Abreu, María Luisa Martín Calvo, Josiana A. Vaz, Ricardo C. Calhelha

**Affiliations:** 1 CIMO, LA SusTEC, Instituto Politécnico de Bragança, Campus de Santa Apolónia, 5300-253 Bragança, Portugal; ana.borges@ipb.pt (A.B.); calhelha@ipb.pt (R.C.C.); 2 Research Center for Active Living and Wellbeing (LiveWell), Instituto Politécnico de Bragança, Campus de Santa Apolónia, 5300-253 Bragança, Portugal; josiana@ipb.pt; 3 Grupo de Investigación en Desarrollo y Evaluación de Formas Farmacéuticas y Sistemas de Liberación Controlada, Facultad de Farmacia, Universidad de Salamanca, Campus Miguel de Unamuno s/n, 37007 Salamanca, Spain; 4 Nutrition and Bromatology Group, Department of Analytical Chemistry and Food Science, Instituto de Agroecoloxía e Alimentación (IAA)—CITEXVI, Universidade de Vigo, 36310 Vigo, Spain; 5 Grupo de Investigación en Fisiología y Farmacología, Facultad de Farmacia, Universidad de Salamanca, Campus Miguel de Unamuno s/n, 37007 Salamanca, Spain; marisam@usal.es

**Keywords:** greater celandine, wound healing, in silico prediction, bioactive natural compounds

## Abstract

Wound healing involves the coordinated regulation of inflammation, angiogenesis, and extracellular matrix remodeling, processes modulated by natural bioactives. In this context, *Chelidonium majus* L. (*C. majus*), a plant rich in alkaloids and flavonoids, remains mechanistically underexplored. This study, therefore, investigates its metabolites using an integrated computational–experimental approach and evaluates their applicability in sericin-based wound-healing systems. A curated database of 83 *C. majus* bioactive compounds was analyzed using cheminformatics and molecular docking against key wound-healing targets (iNOS, VEGF, MMP-3, and tyrosinase), followed by ADMET and toxicity prediction (StopTox). Selected plant–sericin formulations were subsequently evaluated for wound-healing activity using an in vitro fibroblast scratch assay. Docking revealed strong binding affinities for several metabolites, particularly protopine, kaempferol-3-rutinoside, cynaroside, hesperidin, quercetin-3-rhamnosylrutinoside, and vitexin, indicating multi-target modulation across inflammatory, proliferative, and remodeling phases of tissue repair. ADMET and toxicity analyses predicted favorable dermal safety and pharmacokinetic profiles for most compounds. Consistently, in vitro assays demonstrated that *C. majus*–sericin systems had fibroblast migration and wound closure in a concentration- and ratio-dependent manner, with improved healing kinetics observed at 150 µg/mL and for formulations containing higher relative proportions of both components. The experimental outcomes supported the pro-angiogenic and matrix-stabilizing mechanisms predicted in silico. Overall, *C. majus* metabolites exhibit polypharmacological wound-healing activity, supporting their integration into sericin-based systems as a promising strategy for topical therapies.

## 1. Introduction

Wound healing is a highly coordinated biological process that restores tissue structure and function following injury. Whether triggered by trauma, surgical procedures, or chronic underlying conditions, the body initiates a complex cascade of molecular and cellular events aimed at re-establishing tissue integrity. This process unfolds through four continuous and overlapping phases—hemostasis, inflammation, cell proliferation, and tissue remodeling, each governed by tightly regulated interactions among cytokines, growth factors, immune cells, and the extracellular matrix [1,2,3,4,5,6,7]. Although the overall sequence is conserved across different wound types, the relative contributions and efficiencies of each phase vary depending on patient- and wound-specific factors, which can influence progression toward successful closure [1,3,8]. Numerous intrinsic and extrinsic factors—such as persistent inflammation, infection, impaired angiogenesis, and metabolic dysfunction—can disrupt the healing cascade and hinder complete tissue repair. Even minor disturbances in these interconnected pathways may drive the transition from acute to chronic wounds, which often require multifaceted therapeutic interventions to restore functional healing [1,8,9,10,11]. Despite major advancements in wound management, including improved dressings, biomedical materials, and infection control strategies, delayed and non-healing wounds remain a persistent clinical challenge [8,12,13].

Considering these challenges, there is growing interest in developing more effective and holistic therapeutic approaches for wound care. Particular attention has been directed toward natural products due to their inherent biocompatibility, anti-inflammatory and antibacterial activities, cost-effectiveness, and sustainability. Their diverse chemical profiles enable interactions with multiple biological targets, making them promising candidates for supporting different phases of the wound-healing process. As optimal wound management requires comprehensive strategies that address the multifactorial nature of impaired healing, natural compounds have emerged as valuable and versatile resources for the development of innovative wound therapies [1,8,14,15].

Natural products have long been incorporated into wound care due to their broad spectrum of therapeutic properties, including antimicrobial, anti-inflammatory, antioxidant, and tissue-regenerative effects [16,17,18,19,20].

Honey, for example, inhibits bacterial growth through its hypertonic environment while stimulating cytokine and growth-factor production, thereby promoting tissue repair [20]. Aloe vera contributes soothing, anti-inflammatory, and moisturizing benefits that support a favorable healing environment [17,20,21], whereas turmeric—rich in curcumin—accelerates wound closure through its antioxidant and immunomodulatory activities [19,20,21]. Other natural agents such as coconut oil, tea tree oil, lavender oil, calendula, manuka honey, propolis, *Achillea millefolium* (yarrow), and *Centella asiatica* (gotu kola), similarly facilitate healing by maintaining moisture, preventing bacterial colonization, reducing pain and inflammation, promoting granulation tissue, stimulating collagen synthesis, and enhancing angiogenesis. While these substances offer valuable benefits, they should be used cautiously in individuals with sensitivities. Overall, their incorporation into wound-care strategies expands therapeutic options and supports a more holistic approach to wound management [17,18,19,20,22].

This growing interest in nature-based therapeutics has driven the development of innovative wound dressings composed of plant-derived materials that not only protect wounds from infection but also actively promote tissue regeneration. Among the botanicals receiving increased scientific attention is *Chelidonium majus* L. (*C. majus*), a medicinal herb rich in bioactive compounds such as alkaloids, flavonoids, and phenolic acids. These constituents exhibit anti-inflammatory and antibacterial activities, suggesting that *C. majus* extracts may help establish a microenvironment conducive to healing. Although historically less prominent in wound-care applications compared to other botanical agents, its longstanding use in traditional medicine—particularly for dermatological conditions—supports its relevance as a potential natural wound-healing compound [23,24,25,26].

Beyond its implications for wound care, *C. majus* is recognized as one of the most valuable medicinal plants within the Papaveraceae family. Species of the Papaveraceae family are notable for producing a diverse array of pharmacologically active alkaloids, which has attracted significant interest in both biochemistry and pharmacology. *C. majus* is widely distributed across temperate regions of Europe, Asia, and North America, and is characterized by its 50–100 cm height, deeply lobed leaves, yellow four-petaled flowers, elongated capsule-like fruits, and distinctive orange-yellow latex [26,27,28,29,30]. The plant’s rich phytochemical profile includes benzophenanthridine alkaloids, flavonoids, and phenolic acids—bioactive metabolites that have been used for centuries in the treatment of a variety of ailments. These compounds underpin its growing importance in modern phytotherapy and reinforce its potential value in developing innovative wound-healing solutions. With its combination of historical relevance, diverse bioactivity, and strong therapeutic promise, *C. majus* stands out as a compelling candidate for integration into advanced wound-dressing materials and natural healing agents [24,28,30,31,32].

**Figure 1:**
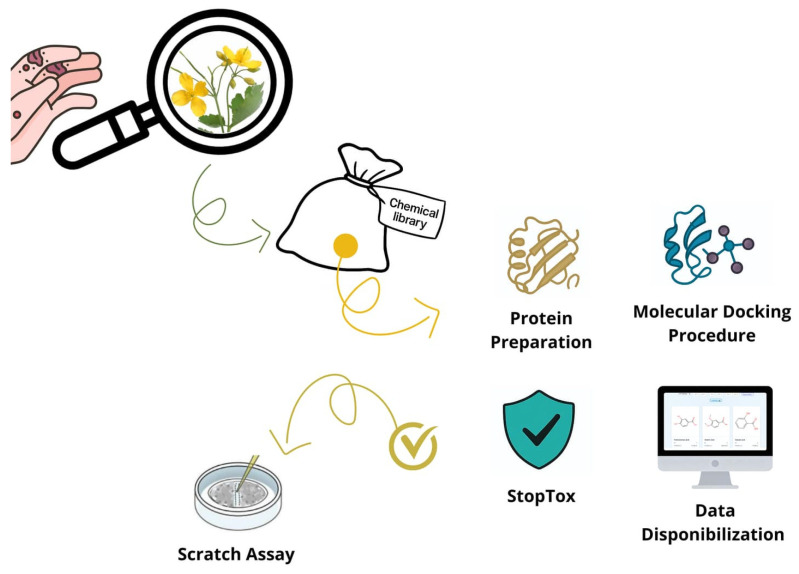
Schematic representation of the integrated in silico and in vitro workflow employed to characterize the chemical diversity, pharmacokinetic properties, molecular interactions, and safety profiles of *Chelidonium majus* L. bioactive compounds in the context of wound healing.

Despite the longstanding use of *C. majus* and other medicinal plants in traditional wound treatments, the molecular mechanisms underlying their therapeutic effects remain insufficiently understood. Current research has largely focused on whole extracts, providing limited insight into how individual bioactive compounds contribute to healing [1]. Furthermore, only a few studies have investigated how these specific molecules interact with key regulatory proteins involved in different stages of wound repair, such as COX-2 and iNOS (inflammation), VEGF (angiogenesis), and matrix metalloproteinases (tissue remodeling). This lack of molecular-level characterization has hindered the ability to establish causative links between the chemical properties of plant-derived compounds and their biological effects. Consequently, a clear gap persists in integrating phytochemical diversity with target-based mechanistic understanding, underscoring the need for systematic approaches that can unravel these interactions more comprehensively [33]. To address these knowledge gaps, combined in silico and in vitro methodologies provide an effective and increasingly adopted strategy. Computational approaches allow rapid screening of large numbers of compounds, predicting physicochemical characteristics, pharmacokinetic behavior, and toxicological risk profiles before experimental validation. Molecular docking and related modeling techniques can identify the most promising interactions between specific plant metabolites and wound-healing targets, thereby narrowing down candidates for laboratory testing. Complementarily, ADMET predictions help reduce experimental cost and minimize safety concerns by filtering out compounds with unfavorable biological profiles [34,35]. When coupled with in vitro assays, these computational insights gain robustness, ultimately enabling a more precise and mechanistic understanding of how *C. majus* metabolites may exert their wound-healing effects. This integrated framework strengthens the translational relevance of the findings and accelerates the early stages of natural-product drug discovery. Accordingly, the present study aimed to elucidate the molecular and pharmacokinetic rationale underlying the wound-healing potential of bioactive compounds from *C. majus* through an integrated in silico and in vitro approach (Figure 1). By combining computational modeling with experimental insights, this work provides a mechanistic foundation that supports the traditional use of *C. majus* and advances its potential application in innovative wound-care therapies.

## 2. Results and Discussion

### 2.1. Physicochemical and Structural Diversity of Bioactive Compounds from Chelidonium majus L.

**Figure 2:**
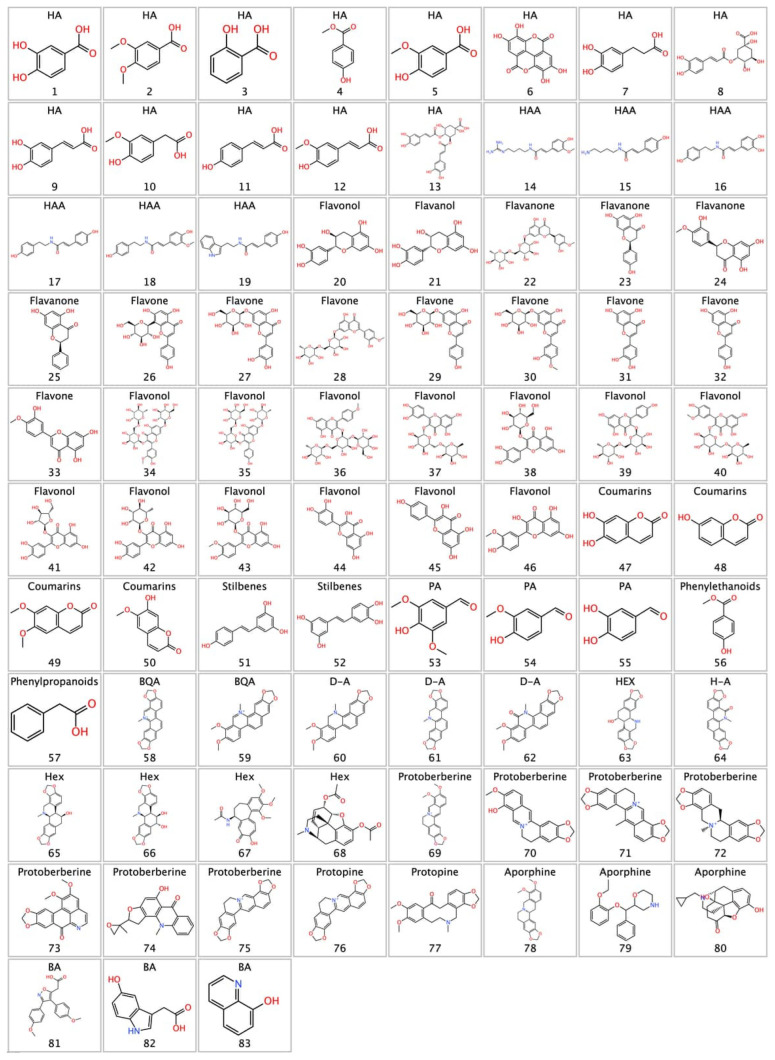
Two-dimensional structures of the 83 bioactive compounds identified in *Chelidonium majus* L. HA: Hydroxy Acids; HAA: Hydroxycinnamic Acid Amides; PA: Phenolic Aldehydes; BQA: Benzophenanthridine Quaternary Amines; D-A: Dihydrobenzophenanthridines; HEX/Hex: Hexahydrobenzophenanthridines; H-A: Oxygenated Benzophenanthridines; BA: Benzophenanthridine Alkaloids.

Natural matrices such as C. *majus* offer a rich reservoir of structurally diverse bioactive compounds, particularly polyphenols and alkaloids. This chemical heterogeneity, characterized by mostly polar structures, with a predominance of aromatic structures functionalized with hydroxyl, methoxyl, carbonyl, and nitrogen-containing functional groups, is shown in Figure 2. The polyphenolic fraction comprises a total of 57 compounds distributed across several structural families. Hydroxybenzoic acids (*n* = 7) and hydroxycinnamic acids (*n* = 6), together with their corresponding amide conjugates (*n* = 6), constitute the simpler phenylpropanoid-derived scaffolds. The flavonoid subclass is the most represented, encompassing flavanols (*n* = 2), flavanones (*n* = 4), flavones (*n* = 8), and flavonols (*n* = 13), the latter being the most abundant group within the entire dataset. Additional phenolic classes include coumarins (*n* = 4), stilbenes (*n* = 2), phenolic aldehydes (*n* = 3), phenyllethanoids (*n* = 1), and phenylpropanoids (*n* = 1). The alkaloid fraction, comprising 27 compounds, is exclusively composed of isoquinoline-derived scaffolds. Benzophenanthridine quaternary alkaloids (*n* = 2), dihydrobenzophenanthridines (*n* = 4), and hexahydrobenzophenanthridines (*n* = 5) represent the benzophenanthridine series in distinct oxidation states. Protoberberines (*n* = 7) constitute the largest alkaloid family, followed by benzophenanthridine alkaloids in their general form (*n* = 3), aporphines (*n* = 3), and protopines (*n* = 2). Together, this diverse ensemble of 83 secondary metabolites reflects the remarkable biosynthetic complexity of *C. majus* and reinforces its relevance as a source of pharmacologically promising molecules.

Understanding this diversity is essential for predicting important molecular properties—such as solubility, permeability, and target affinity—that directly influence pharmacological performance and formulation development. Importantly, the broad spectrum of chemical functionalities present in *C. majus* aligns with the growing interest in designing multicomponent or hybrid biomaterials for wound healing [1]. In this context, its structural diversity suggests potential synergistic interactions with biopolymers such as sericin, a protein widely explored for its moisturizing, anti-inflammatory, and cytoprotective properties [1,13]. This compatibility reinforces the potential to integrate *C. majus* metabolites into sericin-based systems for tissue repair, inflammation modulation, and cell protection during wound healing.

**Figure 3:**
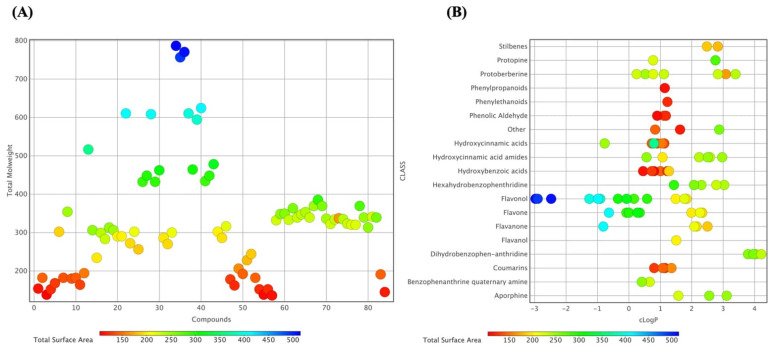
(**A**) Distribution of the molecular weight (MW) of compounds identified in *Chelidonium majus* L. based on total surface area (TSA), showing a predominance of molecules between 200–500 Da and TSA values ranging from 200–400 Å^2^. (**B**) Classification of compounds by chemical class relative to their partition coefficient (cLogP), highlighting a wide variation in lipophilicity among flavonoids, benzophenanthridine alkaloids, and coumarins. The color scale indicates TSA, from red (150 Å^2^) to blue (500 Å^2^).

Analyzing and predicting chemical properties like molecular weight (MW), total surface area (TSA), and lipophilicity (cLogP) is essential for forecasting the biological behavior of bioactive molecules across various chemical scaffolds such as flavonoids, phenolic acids, and benzophenanthridine alkaloids. MW and TSA reflect molecular size and steric complexity, influencing diffusion, target accessibility, and transport across biological barriers [36,37]. In parallel, cLogP is a measure of the balance between aqueous solubility and membrane permeability, shaping absorption, distribution, and metabolic stability [37]. Figure 3 provides an integrated physicochemical overview of the 83 identified compounds. The plot correlates MW with TSA, encoded by a color gradient, revealing a wide distribution from low-molecular-weight phenolic acids (150–250 Da) to high-molecular-weight alkaloids (>700 Da). Polarity is highlighted by classifying compounds by predicted cLogP values: hydrophilic phenolic acids have lower cLogP (<1), whereas lipophilic alkaloids have higher cLogP (>2), suggesting membrane affinity and potential intracellular interactions. Together, these visualizations depict the chemical balance between hydrophilic and lipophilic compounds in *C. majus*, supporting the rationale for subsequent molecular docking studies (Appendix A) against the wound-healing-related targets listed in Table 1.

Importantly, several of the top-ranked compounds identified in Table 1, including protopine (**77**) and multiple flavonoid glycosides, such as rutin (**37**) and quercetin derivatives. Among these, protopine represents a relevant alkaloid component contributing to the plant’s pharmacological profile, while flavonoids such as rutin and quercetin derivatives are associated with antioxidant and tissue-regenerative properties. However, it is important to note that alkaloids such as chelidonine, sanguinarine, and chelerythrine are typically present in higher concentrations in *C. majus* [1,38]. Therefore, although Table 1 highlights compounds with strong predicted binding affinities, their biological relevance should be interpreted by integrating both docking performance and natural abundance within the plant matrix. This combined perspective strengthens the translational relevance of the identified compounds as potential wound-healing agents.

### 2.2. Molecular Docking Analysis of Wound-Healing Targets Across Inflammatory, Proliferative, and Remodeling Phases

A network of enzymatic mediators and growth factors orchestrates the inflammatory, proliferative, and remodeling phases of tissue healing [39]. Understanding how these mediators are related to the wound healing process is necessary to choose strategic targets in molecular docking and virtual screening studies of natural compounds that may heal wounds. COX-2 [40] and iNOS [41] are inflammatory enzymes that are key targets in tissue repair and regulate pro-inflammatory mediators and the initial immune response; Tyrosinase, involved in melanin synthesis and epidermal regeneration after tissue damage [42].

VEGFR-2 is the primary receptor that regulates the formation of new blood vessels by controlling the proliferation, migration, and survival of endothelial cells [43]. MMP-3 is an enzyme that breaks down components of the extracellular matrix, facilitating tissue remodeling, activating other metalloproteinases, and playing a role in inflammation, healing, and the instability of atherosclerotic plaques [44]. The molecular docking data revealed a broad range of binding affinities among the 83 screened compounds across the five wound-healing targets (COX-2, iNOS, VEGFR-2, Stromelysin-1 (MMP-3), and Tyrosinase), with values spanning approximately −4.0 to −11.6 kcal/mol. This variation indicates different molecular interaction profiles, suggesting differences in structural compatibility and binding strength among the tested molecules. The inflammatory targets (COX-2 and iNOS) showed a consistent pattern, with several compounds significantly outperforming the control ligand across all enzymes.

**Figure 4:**
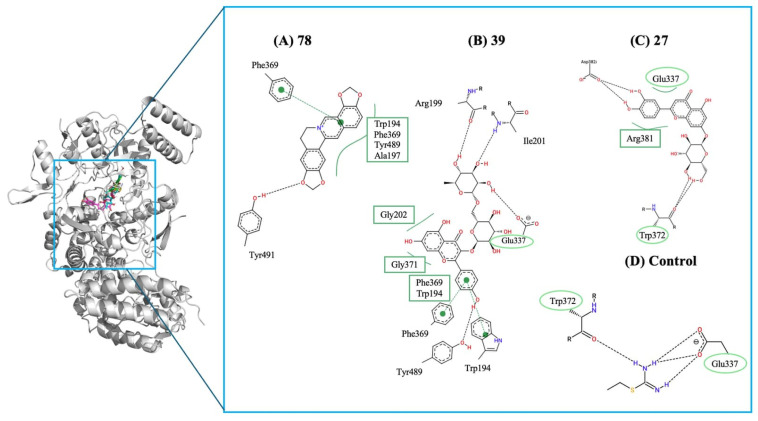
Predicted binding interactions of the top iNOS inhibitors compared with the control ligand (Ethylisothiourea). The 3D overview (left) displays the docking poses of the ligands within the iNOS active site, represented by a grey ribbon structure. Panels (**A**–**C**) display the 2D interaction maps for protopine (**78**), kaempferol 3-rutinoside (**39**), and cynaroside (**27**), respectively, while panel (**D**) shows the control ligand. In the 2D maps, black dashed lines indicate classical hydrogen bonds, and green arcs denote hydrophobic interactions. Green dashed lines with central nodes represent Pi-type interactions (such as Pi-Pi or Pi-alkyl). Residues highlighted with green circles or ovals indicate binding interactions that are shared with the control ligand, demonstrating a conserved inhibitory profile and validating the binding mode of the most promising compounds within the catalytic pocket.

For COX-2, mefenamic acid (control ligand) showed a predicted binding energy of –10.2 kcal/mol. None of the screened compounds matched this affinity level. Although several molecules were predicted to interact within the active site, all had weaker binding energies than the reference inhibitor, probably indicating limited potential for this library to inhibit COX-2 effectively. Conversely, a different pattern was evident for iNOS. While ethylisothiourea (control ligand) bound at −5.6 kcal/mol, multiple compounds displayed significantly stronger predicted affinities (Figure 4). Notably, protopine (**78**) exhibited a predicted binding energy of −11.6 kcal/mol, the strongest interaction observed across the dataset. Kaempferol 3-rutinoside (**39**) and cynaroside (**27**), with energies of −11.4 kcal/mol and −11.2 kcal/mol, respectively, also performed remarkably well. However, it is important to interpret these scores in the context of molecular size. The significantly better binding energies observed for these plant metabolites compared to ethylisothiourea are expected, as larger ligands inherently form more intermolecular contacts, inflating the absolute docking scores. When considering Ligand Efficiency (LE) [45], which normalizes the binding energy by the number of heavy atoms (LE = ΔG/NHA), the small control ligand (6 heavy atoms) exhibits an outstanding LE of approximately −0.93 kcal/mol/HA. In contrast, the larger plant metabolites show lower per-atom efficiencies: protopine (**78**) (26 heavy atoms, LE ≈ −0.45 kcal/mol/HA), cymaroside (**27**) (32 heavy atoms, LE ≈ −0.35 kcal/mol/HA), and kaempferol-3-rutinoside (**39**) (42 heavy atoms, LE ≈ −0.27 kcal/mol/HA). This demonstrates that while compounds **78**, **39**, and **27** effectively occupy and stabilize the iNOS active site, their superior absolute affinities reflect characteristic large-scaffold interactions rather than disproportionately high per-atom efficiencies.

These values, nearly twice those of the control, suggest a strong connection to the iNOS catalytic pocket and identify these molecules as promising candidates for modulating nitric oxide–driven inflammatory signaling. Overall, these interactions account for the significantly stronger binding affinities of compounds **78**, **39**, and **27** relative to the control. These promising molecules enhance the stabilization of critical inflammatory targets and demonstrate highly favorable scores. The results, which surpass the power, are linked to modulation of nitric oxide pathways, a key mechanism in regulating inflammation during the initial phase of wound healing [46,47,48]. Figure 4 displays the 2D interaction maps for compounds (A) protopine (**78**), (B) Kaempferol 3-rutinoside (**39**), and (C) cynaroside (**27**). It highlights key hydrogen bonds, π–π stacking interactions, and hydrophobic contacts with residues such as Trp194, Phe369, Tyr491, Gly203, and Ala317. Additionally, it shows the interaction profile of the control ligand, which has fewer stabilizing contacts and lacks the extensive aromatic stacking seen in the most effective compounds.

**Figure 5:**
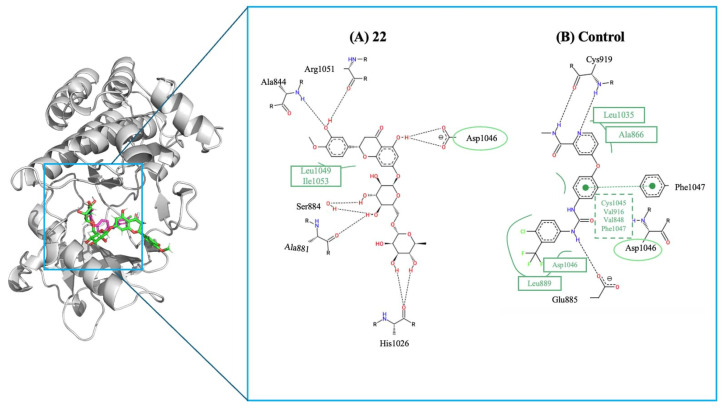
Comparison of the binding interactions of the top VEGFR-2 compound compared to the control ligand. The 3D overview (left) illustrates the docking poses of hesperidin (**22**, green sticks) and the control ligand (sorafenib, pink sticks) within the VEGFR-2 catalytic pocket, represented by grey ribbons. Panels display the 2D interaction maps for (**A**) hesperidin (**22**) and (**B**) the control ligand (sorafenib). In the 2D maps, black dashed lines represent classical hydrogen bonds (e.g., N-H or O-H interactions), while green arcs denote hydrophobic contacts. The green dashed lines with central nodes in panel (**B**) indicate Pi-type interactions (e.g., Pi-stacking or Pi-alkyl). Residues highlighted with green circles or ovals (e.g., Asp1046) indicate amino acids that are shared with the control ligand’s binding profile, demonstrating that compound **22** interacts with key conserved regions of the VEGFR-2 active site.

During the proliferative phase, VEGFR-2 highlighted distinct groups of compounds with higher predicted inhibitory potential than their respective controls. For VEGFR-2, the key regulator of angiogenesis during this stage, which exhibited a sorafenib (control) affinity of −9.3 kcal/mol (Figure 5B), although none of the screened compounds surpassed this value, several emerged as top interactors within the library. The most notable was compound hesperidin (**22**), reaching −9.4 kcal/mol (Figure 5A). While these affinities do not exceed the control ligand, they fall within the upper range of predicted VEGF interactions and suggest meaningful engagement with the VEGFR-2 binding interface. Such interactions support their potential roles in promoting endothelial activation, microvascular sprouting, and early extracellular matrix deposition. The 2D interaction maps reveal hydrogen bonds, hydrophobic contacts, and key stabilizing interactions involving residues like Arg513, Ser530, Leu352, and Ala516. Although both ligands bind to similar areas within the pocket, the control form establishes more extensive hydrophobic contacts with residues Phe381, Phe518, and Tyr385, which boost its binding affinity.

**Figure 6:**
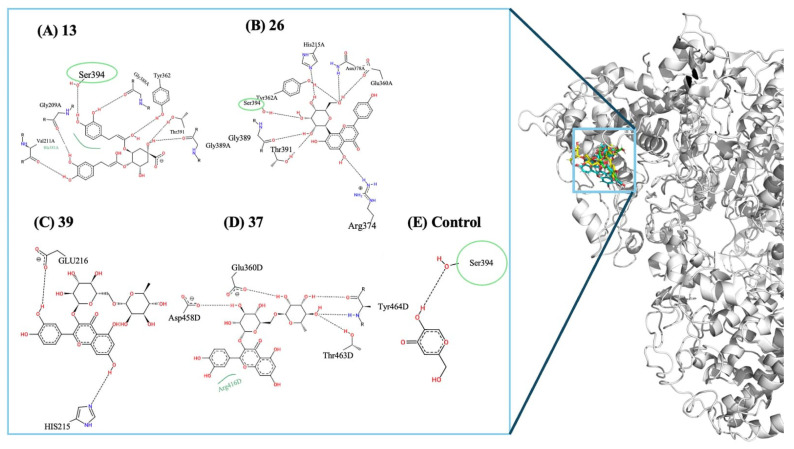
Predicted binding interactions of the top tyrosinase-binding compounds compared with the control ligand. The 3D illustration (right) emphasizes the docking poses within the tyrosinase catalytic pocket. Panels (**A**–**D**) display the 2D interaction maps for compounds 4,5-dicaffeoylquinic acid (**13**), Vitexin (**26**), kaempferol 3-rutinoside (**39**), and Rutin (**37**), respectively, while panel (**E**) represents the control ligand. In the 2D interaction maps, black dashed lines indicate classical hydrogen bond interactions, and green arcs denote hydrophobic contacts. The green circles or ovals (e.g., Ser394) highlight the key residues that are shared with the control ligand’s binding profile, demonstrating a conserved inhibitory pattern and validating the binding mode of the most promising compounds within the active site.

As the healing process advances into the remodeling phase, two additional targets, stromelysin-1 (MMP-3) and tyrosinase, also revealed sets of compounds with significantly stronger predicted affinities than their respective controls. For MMP-3, a key metalloproteinase involved in extracellular matrix turnover, the hydroxamate-based inhibitor (control ligand) showed a binding energy of −6.2 kcal/mol. Four compounds, shown in Figure 6, significantly improved interactions, with diosmin (**28**) (−10.2 kcal/mol) leading, followed by pinocembrin (**25**) and quercetin-3-rhamnosylrutinoside (**35**), both around −9.6 kcal/mol. These enhanced affinities suggest a strong ability to stabilize the MMP-3 catalytic pocket, indicating that these molecules may help regulate matrix degradation, supporting balanced tissue remodeling and proper scar maturation.

**Figure 7:**
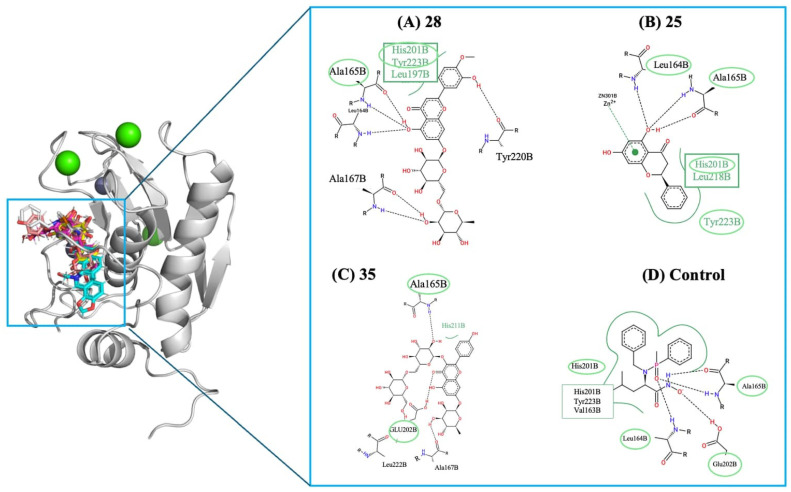
Predicted binding interactions of the top MMP-3 inhibitors compared to the control ligand. The 3D overview (left) depicts the docking poses within the MMP-3 catalytic cleft; the grey sphere represents the catalytic Zn^2+^ ion, while green spheres represent structural Ca^2+^ ions. Panels (**A**–**C**) display the 2D interaction maps for compounds Diosmin (**28**), Pinocembrin (**25**), and Quercetin-3-rhamnosylrutinoside (**35**), respectively, and panel (**D**) shows the control ligand. In the 2D maps, black dashed lines indicate classical hydrogen bonds (e.g., involving N-H groups from the protein backbone), green dashed lines represent metal-coordination with the zinc ion, and green arcs denote hydrophobic interactions. Residues highlighted with green circles/ovals indicate binding interactions that are common to both the respective compound and the control ligand, emphasizing the conservation of key inhibitory contacts within the active site.

A similar trend was seen for tyrosinase, a critical enzyme in melanogenesis and post-inflammatory pigmentation during late remodeling. The kojic acid (control) were predicted to bound at −5.8 kcal/mol, while several compounds showed more favorable energies (Figure 7), especially 4,5-dicaffeoylquinic acid (**13**) (−8.1 kcal/mol) and vitexin (**26**) (−7.8 kcal/mol), with kaempferol 3-rutinoside (**39**) and Rutin (**37**), clustering around −7.7 kcal/mol. These improved interactions indicate better predicted binding ability of the tyrosinase active site and highlight the potential for these molecules to influence pigmentation pathways, potentially reducing post-inflammatory hyperpigmentation and enhancing scar appearance. These molecules engage in multiple hydrogen bonds and polar contacts with critical residues such as Ser394, Arg308, Asp420, and Ser728, while further stabilizing the active site through additional hydrophobic and π-stacking interactions. In comparison, the control ligand exhibits a simpler interaction pattern, primarily forming a single hydrogen bond with Ser394. The broader interaction networks seen in compounds **13**, **26**, **39**, and **37** help clarify their higher predicted affinities.

Finally, Figure 7 presents the 2D interaction maps for compounds Diosmin (**28**), Pinocembrin (**25**), and Quercetin-3-rhamnosylrutinoside (**35**), illustrating key hydrogen bonds, polar contacts, and hydrophobic interactions with residues like Asp192, His201, Tyr155, Leu164, and Ala165. These compounds also establish crucial metal-coordination interactions with the catalytic zinc ion, suggesting they effectively occupy and inhibit the active site. In contrast, the control ligand exhibits a more limited contact network with fewer interactions and decreased overall stabilization. The broader interaction profiles of compounds Diosmin (**28**), Pinocembrin (**25**), and Quercetin-3-rhamnosylrutinoside (**35**) support their higher predicted affinities for MMP-3. Nevertheless, the limitations of molecular docking must be acknowledged. Docking relies on a static receptor model that neglects protein conformational flexibility upon ligand binding, and its scoring functions provide only approximate estimates of binding affinity, failing to fully account for solvation effects, entropic contributions, and the explicit role of water molecules at the binding interface. Consequently, the predicted affinities should be interpreted as relative rankings rather than absolute thermodynamic values. However, the experimental wound-healing assays presented in Section 2.4 provide biological validation that partially corroborates the computational findings, strengthening the overall reliability of the results obtained for the most promising compounds.

### 2.3. Toxicological Risk Assessment and Selection of Non-Sensitizing Candidates

The selected compounds, according to the prediction models tested, showed low acute inhalation and oral toxicity and did not cause significant eye irritation, as demonstrated in Table 1. The key factor was skin sensitization, a critical safety concern for formulations. Only compound pinocembrin (**25**) posed a risk of causing allergic reactions and should therefore be excluded. Compounds hesperidin (**22**), vitexin (**26**), and quercetin-3-rhamnosylrutinoside (**35**) demonstrated the strongest safety profiles, consistently classified as non-sensitizers with high confidence (80%), making them the most promising candidates. Structurally, all three belong to the flavonoid class: hesperidin (**22**) is a flavanone glycoside bearing a methoxylated B-ring and a rutinoside at position 7; vitexin (**26**) is a flavone C-glycoside with a glucose unit directly attached at position 8 of the apigenin scaffold [49]; and quercetin-3-rhamnosylrutinoside (**35**) is a flavonol O-glycoside with a polyhydroxylated B-ring and a branched trisaccharide at position 3. These glycosidic substituents, combined with their polyhydroxylated aromatic cores, confer high polarity and molecular stability, likely contributing to their favorable toxicological profiles. Compounds cynaroside (**27**), diosmin (**28**), 4,5-dicaffeoylquinic acid (**13**), and rutin (**37**) are also safe, but with moderate confidence. Therefore, hesperidin (**22**), vitexin (**26**), and quercetin-3-rhamnosylrutinoside (**35**) should be prioritized for further development, as their predicted safety profiles are strongly supported by experimental evidence in the literature.

Hesperidin has been widely reported to have anti-inflammatory, antioxidant, antimicrobial, and pro-healing effects on the skin. Studies show that topical hesperidin accelerates wound healing, improves collagen organization, and protects skin tissues from stressors such as UV radiation or irradiation injury, confirming its importance for both the inflammatory and proliferative stages of tissue repair [50]. Vitexin shows significant potential in dermatology, demonstrating vigorous antioxidant activity and protecting human dermal fibroblasts from UVB-induced oxidative damage. This indicates its possible role in reducing early oxidative stress and promoting fibroblast health during the proliferative phase [51]. Finally, quercetin derivatives, including quercetin-3-rhamnosylrutinoside, are well-known for their role in skin regeneration. Reviews and in vivo studies indicate that these compounds suppress inflammatory mediators such as COX-2 and iNOS, promote collagen production and fibroblast activity, encourage the formation of new blood vessels, and can even reduce post-inflammatory hyperpigmentation by inhibiting tyrosinase [52]. These findings confirm that compounds **22**, **26**, and **35** exhibit roles in controlling inflammation, promoting tissue regeneration, reducing oxidative stress, and improving scar quality, as indicated by computational studies. Consequently, they are the most promising candidates for topical wound-healing treatments and warrant further experimental validation.

### 2.4. Wound-Healing Activity

All tested conditions showed a progressive reduction in wound area from day 0 to day 4. Across both concentrations, the decrease in wound area was particularly evident between days *t* = 2 and *t* = 4, and several formulations approached the healing profile of the positive control (allantoin) while outperforming the negative control (culture medium DMEM), showing biologically relevant pro-migratory activity. However, the magnitude of closure varied considerably between formulations, indicating that the wound-healing response depended strongly on sericin source and plant–sericin ratio rather than occurring uniformly across all samples.

**Figure 8:**
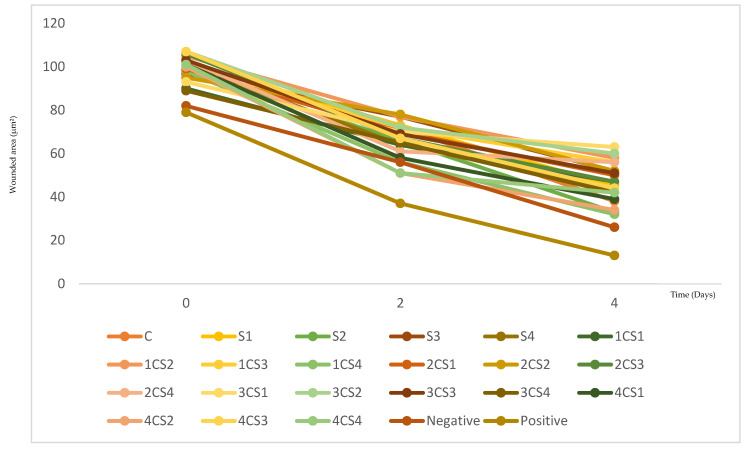
Wound healing activity of the different samples studied. Results were analyzed by the photographs of the wounded area at *t* = 0; *t* = 2, and *t* = 4, at the concentrations of 100 μg/mL: S1—Sericin from Castelo Branco; S2—Sericin from Bragança; S3—Sericin purchased from Sigma-Aldrich; S4—Sericin purchased from FUJIFILM Wako Chemicals; C—*C. majus*. 1CS—Ratio 1 *C. majus:* 1 Sericin (200:1600 µg/mL); 2CS—Ratio 1 *C. majus*: 2 Sericin (200:3200 µg/mL); 3CS—Ratio 2 *C. majus*: 1 Sericin (400:1600 µg/mL); 4CS—Ratio 2 *C. majus*: 2 Sericin (400:3200 µg/mL). The controls were analyzed at the concentration of 400 μg/mL: (^−^C) Negative control: Culture medium; (^+^C) Positive control: Allantoin (10 mg/mL).

**Figure 9:**
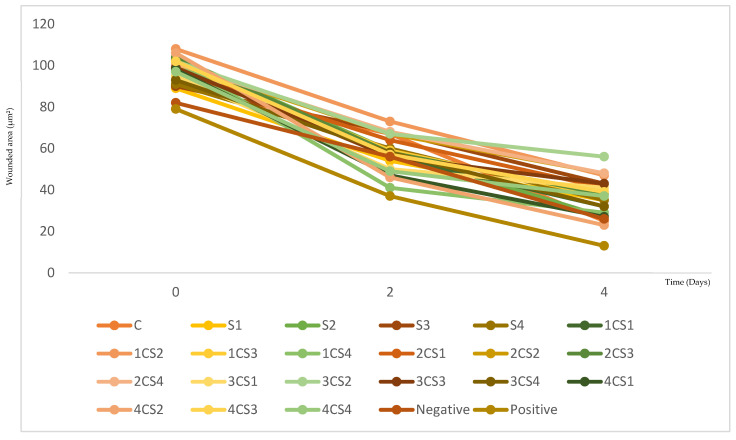
Wound healing activity of the different samples studied. Results were analyzed by the photographs of the wounded area at *t* = 0; *t* = 2, and *t* = 4, at the concentrations of 150 μg/mL: S1—Sericin from Castelo Branco; S2—Sericin from Bragança; S3—Sericin purchased from Sigma-Aldrich; S4—Sericin purchased from FUJIFILM Wako Chemicals; C—*C. majus*. 1CS—Ratio 1 *C. majus:* 1 Sericin (200:1600 µg/mL); 2CS—Ratio 1 *C. majus*: 2 Sericin (200:3200 µg/mL); 3CS—Ratio 2 *C. majus*: 1 Sericin (400:1600 µg/mL); 4CS—Ratio 2 *C. majus*: 2 Sericin (400:3200 µg/mL). The controls were analyzed at the concentration of 400 μg/mL: (^−^C) Negative control: Culture medium; (^+^C) Positive control: Allantoin (10 mg/mL).

For clarity, the abbreviations used throughout this section are defined as follows: “C” represents *C. majus* extract, “S” corresponds to sericin samples (from different origins), and “CS” refers to plant–sericin combinations. The numerical identifiers (e.g., CS1, CS2, S2, S3) correspond to specific formulations and ratios, as detailed in Figure 8 and Figure 9.

At 100 µg/mL (formulation concentration), differences among formulations became clearer over time (Figure 8). Between day 0 and day 2, most samples showed only moderate reductions in wound area. Still, some combinations already displayed accelerated closure, most notably 4CS2 and 4CS4, which surpassed the negative control at day 2, while 1CS4 and 4CS1 followed closely. Conversely, several combinations, such as 1CS2, 2CS2, and S3 maintained comparatively higher wound areas at day 2, indicating slower early migration despite eventual closure. By day 4, the strongest wound closure was observed for 1CS4, followed by S2 and 4CS2, whereas 3CS1 and 3CS2 remained among the least effective, retaining larger residual wound areas.

At 150 µg/mL (formulation concentration), the overall healing response was more pronounced and the separation between better- and weaker-performing systems became clearer across timepoints (Figure 9). At day 2 some combinations surpassed the negative control. Among those, 1CS4 emerged as the most responsive condition, close to the positive control, with 4CS2 and 4CS1 also showing faster wound-area reduction relative to other formulations. In contrast, the combination 1CS2 still presented a higher residual wound area, indicating that increasing concentration did not uniformly enhance all ratios. By day 4, 4CS2 achieved the lowest residual wound area among the combinations and was closely followed by the plant extract alone (*C. majus*) as one of the strongest performers at this concentration. Nevertheless, some formulations continued to exhibit comparatively higher wound areas, confirming that the pro-migratory effect remained dependent on composition rather than concentration alone.

Taken together, 150 µg/mL provided the best overall performance, producing greater and earlier wound-area reduction across most conditions. These concentrations were selected within previously validated cytocompatible ranges for the same *C. majus*-sericin systems [38], ensuring that the observed effects are associated with enhanced cell migration rather than cytotoxic responses.

Considering both concentrations and temporal progression, the most effective combination was 4CS2 (combination of 400 *C. majus*+ 3200 sericin µg/mL; Bragança sericin) at 150 µg/mL, which consistently exhibited one of the lowest residual wound areas and the most advanced wound-closure kinetics among the tested plant–sericin systems.

These findings align with previous studies, which [53] reported that sericin-based wound dressings enhance cell migration, which is essential for treating severe skin injuries and improving healing outcomes, particularly in large-area wounds. Similarly, ref. [54] demonstrated that aqueous *C. majus* extract promoted wound closure by eradicating infections, exhibiting anti-inflammatory effects, and supporting re-epithelialization. The observed synergy between *C. majus* and Sericin highlights their potential for wound healing applications, emphasizing the importance of optimizing specific combinations for enhanced therapeutic effects.

The experimental findings obtained from the scratch assay can be mechanistically correlated with the in silico docking results presented in Section 2.2. The compounds identified as top docking candidates—particularly alkaloids such as protopine and flavonoid derivatives including kaempferol glycosides and quercetin-related compounds—exhibited strong binding affinities toward key wound-healing targets, namely iNOS, VEGFR-2, MMP-3, and tyrosinase. These targets are directly involved in the regulation of inflammation, angiogenesis, and extracellular matrix remodeling, which are critical processes governing fibroblast migration and wound closure.

The enhanced wound-healing activity observed in the in vitro assays, particularly for selected plant–sericin combinations, is consistent with this multi-target modulation. In this context, the improved wound-closure kinetics observed for the most effective formulations (e.g., 4CS2) may be explained by the combined action of multiple bioactive compounds acting on different stages of the healing cascade.

Importantly, these results suggest a synergistic effect arising from both the chemical diversity of *C. majus* and its integration within the sericin matrix. While individual compounds contribute through specific molecular interactions, their combined presence enables a polypharmacological response, simultaneously targeting multiple biological pathways. In addition, sericin itself provides a favorable microenvironment that enhances cell adhesion and migration, thereby potentiating the biological activity of plant-derived metabolites.

Despite these promising results, some limitations associated with the in vitro scratch assay model should be considered. This model primarily evaluates cell migration in a simplified two-dimensional environment and does not fully replicate the complexity of in vivo wound healing, which involves additional processes such as immune response, vascularization, and extracellular matrix remodeling in a three-dimensional context. Furthermore, the assay does not distinguish between the contributions of cell proliferation and migration with complete accuracy. Therefore, while the present findings provide valuable insights into the wound-healing potential of *C. majus*–sericin systems, further studies using more advanced models, including 3D skin equivalents and in vivo assays, are required to fully validate their therapeutic applicability.

## 3. Materials and Methods

### 3.1. Bioactive Molecules Preparation

The target proteins (Table 2) were obtained from the Protein Data Bank (PDB) in their co-crystallized forms and prepared within the SAMSON environment [55]. During preparation, water molecules and non-essential ligands were removed, leaving only the cofactors and all residues of each of the selected protein structures. Subsequently, each structure was protonated and energy-minimized to ensure conformational stability before docking simulations. The resulting proteins were saved in .pdbqt format, with active sites defined from the coordinates of the native ligands, ensuring accurate molecular docking using VINA [56]. To validate the docking protocol, all targets underwent redocking procedures in which the co-crystallized ligand was re-docked into its respective binding site, confirming the reliability of the grid parameters and scoring function. The calculated RMSD values for each target are summarized in Table A2. During preparation, water molecules and non-essential ligands were removed, leaving only the cofactors and all residues of each of the selected protein structures. Subsequently, each structure was protonated and energy-minimized to ensure conformational stability before docking simulations. The resulting proteins were saved in .pdbqt format, with active sites defined from the coordinates of the co-crystallized ligands, ensuring accurate molecular docking using VINA [56]. To validate the docking protocol, all targets underwent redocking procedures in which the co-crystallized (Table 2) ligand was re-docked into its respective binding site, confirming the reliability of the grid parameters and scoring function.

### 3.2. Molecular Docking Procedure

Molecular docking simulations were performed using AutoDock VINA 1.2.0 [56] to predict the binding affinity and most favorable orientations of each ligand within the active site of the selected target proteins. All ligands were previously minimized in energy, had their Gasteiger charges assigned, and were converted to .pdbqt format to ensure compatibility with the scoring function. For each protein, the docking search space was defined based on the coordinates of the co-crystallized ligand, allowing the grid box to completely encompass the native binding site while providing sufficient flexibility for ligand exploration; the grid dimensions are 30 Å on each axis. The docking simulations were run through the SAMSOM v6.0.0 software interface. All molecular docking results were visually inspected using SAMSON and PyMOL 3.1 to verify pose plausibility, confirm appropriate interactions within the active site, and assess the presence of hydrogen bonds, hydrophobic contacts, or π–π interactions commonly associated with the expected ligand mechanism of action. Furthermore, qualitative analysis of ligand-protein interactions was performed using the ProteinPlus platform [61]. The entire molecular docking workflow was systematically applied to all compounds, ensuring methodological consistency and robustness in the comparative interpretation of binding affinities. 

### 3.3. Stoptox Avability

Toxicological screening was conducted using the StopTox platform, a machine-learning-based tool developed from curated toxicological databases aligned with the Globally Harmonized System (GHS). StopTox combines information from ACToR, Tox21, ToxCast, REACH, and other regulatory collections to generate QSTR models capable of predicting multiple acute toxicity endpoints. After molecular docking analyses, all compounds were processed in StopTox using their canonical SMILES to ensure structural consistency. The platform provided toxicity classifications for acute oral, inhalation, and dermal toxicity, as well as predictions for eye irritation, skin irritation, and skin sensitization, with confidence scores expressed as percentages. Only molecules classified as “Non-Toxic (−)” or deemed low-risk within GHS criteria were retained for subsequent ADMET interpretation, while compounds flagged with high-risk alerts (GHS categories 1–3) were excluded. This filtering step ensured that only candidates with both favorable binding affinities and acceptable toxicological profiles advanced to the pharmacokinetic and safety analyses, enhancing the robustness and translational relevance [62].

### 3.4. Development of the Database

The *Chelidonium majus* L. Database platform was developed using a Serverless Static Site architecture hosted on GitHub https://cshiraishi.github.io/CMD/ (accessed on 1 April 2026), prioritizing performance and security through the use of pre-processed data over dynamic relational databases. The chemoinformatics curation and processing pipeline was automated in Python (3.x), using the pandas library for the initial structuring of the data and the RDKit [63] package for validating 83 SMILES codes [1,38], calculating essential physicochemical descriptors (molecular weight, LogP, and TPSA), and generating vector representations (SVG) and structural files (.sdf) for molecular docking studies. The system compiles this information into a static JSON object that feeds a user interface built with native web technologies (HTML5, CSS3 and JavaScript ES6+), which integrates the Chart.js library for visual statistical analysis and allows real-time client-side filtering and interaction.

The compounds included in the *C. majus* database were compiled from a combination of literature-reported phytochemical studies and our previous experimental work [38], in which the plant’s chemical profile was characterized. The dataset comprises 83 bioactive molecules representing the main chemical classes described for *C. majus*, including benzophenanthridine alkaloids, flavonoids, phenolic acids, and coumarins.

Among these, several compounds—such as chelidonine, sanguinarine, chelerythrine, protopine, berberine, and coptisine—are well-documented as major alkaloid constituents of *C. majus*, particularly in the latex, and are known to contribute significantly to its biological activity. Additionally, flavonoids such as rutin, quercetin derivatives, and kaempferol glycosides have been reported, although generally at lower concentrations than alkaloids [1].

This distinction between major and minor constituents is essential for interpreting the docking results, as compounds present at higher abundance are more likely to contribute to the plant’s overall pharmacological effects and those of its derived formulations.

### 3.5. In Vitro Wound-Healing Potential

The biological relevance of *C. majus* extracts, sericin biomaterial, and their combined systems has previously been investigated by our group about their suitability for biomedical and topical applications [1,38,64]. Experimental procedures involving plant extraction, combination with sericin, and formulation development have been described in [38], where *C. majus* extracts and their integration with sericin were investigated. In addition, the sericin samples used in this study correspond to those previously isolated and characterized in [64], and are identified according to their origin to ensure consistency with the reported formulations and their biological evaluation. These prior findings provide contextual experimental support for the translational relevance of the present computational screening, establishing a foundation for exploring the wound-healing potential of bioactive compounds identified in silico.

To provide experimental validation directly aligned with the molecular predictions generated in this study, additional in vitro evaluation was performed through a scratch wound migration assay.

The cell migration scratch assay followed the protocol outlined by [65]. Initially, Human fibroblast cells (HFF1), obtained from the Leibniz Institute DSMZ—German Collection of Microorganisms and Cell Cultures GmbH (Braunschweig, Germany), were cultured in 26-well plates until confluency. The DMEM medium was aspirated upon confluence, and a scratch was gently created in the cell monolayer using a plastic pipette tip (100 μL). Subsequently, the cells were washed with HBSS to eliminate any detached cells, and the culture medium containing the extracts at various concentrations was added. The concentrations tested were determined based on results from assessing the extracts’ toxicity to fibroblasts. The culture medium served as the negative control, while allantoin (10 mg/mL), a known wound-healing agent, was used as the positive control. Photographs of the wounded area were captured at both *t* = 0 and *t* = 4 days using a phase-contrast microscope (Nikon Eclipse TS100, Nikon Corporation, Tokyo, Japan) equipped with a digital camera (Carl Zeiss 4 Studio Co., Ltd. Axiocam 208 Color, Zeiss, Oberkochen, Germany). These images were then analyzed using Image J software with the MRI Wound Healing tool to determine the extent of wound closure over time.

The concentrations tested (100 and 150 µg/mL) were selected based on previously published results from our group, in which the same *C. majus*–sericin samples were evaluated for cytocompatibility using antiproliferative assays in human skin fibroblasts [39]. These concentrations were demonstrated to be non-cytotoxic, ensuring that the effects observed in the scratch assay are primarily associated with cell migration rather than cytotoxicity or proliferation inhibition.

## 4. Conclusions

This study integrated molecular modeling, docking, and toxicological predictions to elucidate the wound-healing potential of the bioactive compounds of *Chelidonium majus* L. The results demonstrated a remarkable diversity of chemical scaffolds, including flavonoids, phenolic acids, and benzophenanthridine alkaloids, each exhibiting distinct physicochemical profiles that contribute to a balanced distribution of hydrophilicity and lipophilicity. Such structural diversity provides the molecular basis for the plant’s multifunctional pharmacological activity and supports its traditional use in topical healing formulations. The in silico docking analyses revealed that several compounds displayed strong and specific interactions with key protein targets involved in the inflammatory (iNOS), proliferative (VEGF), and remodeling (MMP-3 and tyrosinase) phases of tissue repair, with binding affinities reaching up to −11.6 kcal/mol (iNOS), compared to −5.6 kcal/mol for the reference ligand. Notably, protopine (**78**), kaempferol 3-rutinoside (**39**), and cynaroside (**27**) emerged as potent iNOS binders, while hesperidin (**22**), quercetin-3-rhamnosylrutinoside (**35**), and vitexin (**26**) demonstrated favorable polypharmacological profiles across multiple wound-healing targets. These findings highlight the synergistic potential of these natural molecules to modulate inflammation, stimulate angiogenesis, stabilize the extracellular matrix, and balance pigmentation. The toxicity screening via StopTox reinforced the biocompatibility of most compounds, identifying hesperidin, vitexin, and quercetin-3-rhamnosylrutinoside as safe and non-sensitizing candidates suitable for dermal application. Importantly, these computational predictions were supported by in vitro scratch-assay results, which confirmed that *C. majus*-sericin formulations promote fibroblast migration and wound closure in a concentration- and ratio-dependent manner. All tested systems reduced wound area over time, with enhanced healing kinetics observed particularly at 150 µg/mL. Among the evaluated formulations, combinations containing higher relative proportions of both components (notably the 4CS2 combination) exhibited the most pronounced closure dynamics, corresponding to the lowest residual wound area among all tested conditions at this concentration, demonstrating a synergistic interaction between plant-derived bioactives and the sericin matrix. These findings experimentally substantiate the predicted modulation of angiogenic and extracellular-matrix-related pathways suggested by the docking analyses. Collectively, the integration of in silico molecular modeling with experimental migration assays provides a comprehensive pharmacokinetic and mechanistic rationale for the therapeutic use of *C. majus* metabolites in regenerative dermatology. In this context, sericin-based wound dressings enriched with *C. majus* bioactives emerge as a promising bioinspired strategy, combining the moisturizing, film-forming, and cytoprotective properties of sericin with the multifunctional biological activity of plant metabolites. Future work should focus on advanced formulation optimization and in vivo validation to develop next-generation topical systems for accelerated tissue repair and improved scar quality.

## Figures and Tables

**Table 1:** Predicted toxicological assessment of selected bioactive compounds.

Compound	Structure	Name	Acute Inhalation Toxicity	Confidence	Acute Oral Toxicity	Confidence	Eye Irritation and Corrosion	Confidence	Skin Sensitization	**Confidence**
**13**	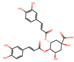	4,5-Dicaffeoylquinic acid	Non-Toxic (−)	72.0%	Non-Toxic (−)	68.0%	Non-Toxic (−)	60.0%	Non-Sensitizer (−)	60.0%
**22**	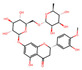	Hesperidin	Non-Toxic (−)	68.0%	Non-Toxic (−)	58.0%	Non-Toxic (−)	69.0%	Non-Sensitizer (−)	80.0%
**25**	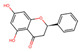	Pinocembrin	Non-Toxic (−)	68.0%	Non-Toxic (−)	69.0%	Non-Toxic (−)	58.0%	Sensitizer (+)	50.0%
**26**	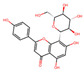	Vitexin	Non-Toxic (−)	76.0%	Non-Toxic (−)	75.0%	Non-Toxic (−)	59.0%	Non-Sensitizer (−)	80.0%
**27**	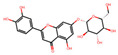	Cynaroside	Non-Toxic (−)	77.0%	Non-Toxic (−)	66.0%	Non-Toxic (−)	70.0%	Non-Sensitizer (−)	70.0%
**28**	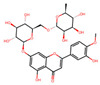	Diosmin	Non-Toxic (−)	68.0%	Non-Toxic (−)	58.0%	Non-Toxic (−)	69.0%	Non-Sensitizer (−)	70.0%
**35**	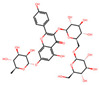	Quercetin-3-rhamnosylrutinoside	Non-Toxic (−)	77%	Non-Toxic (−)	69.0%	Non-Toxic (−)	75.0%	Non-Sensitizer (−)	80.0%
**37**	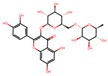	Rutin	Non-Toxic (−)	70.0%	Non-Toxic (−)	70.0%	Non-Toxic (−)	74.0%	Non-Sensitizer (−)	60.0%

**Table 2:** Human (and homologous) protein targets involved in wound healing, co-crystallized with reference inhibitors.

Target	PDB Code	Co-Crystallized	Coordinates	Organism	Classification	References
Cyclooxygenase-2 (COX-2)	5IKR	Mefenamic Acid	X = 39.810 Y = 1.943 Z = 63.116	*Homo sapiens*	Oxidoreductase	[57]
Inducible nitric oxide synthase (iNOS)	4NOS	Ethylisothiourea	X = −2.00 Y = 97.670 Z = 21.297	*Homo sapiens*	Oxidoreductase	[58]
Vascular endothelial growth factor receptor 2 (VEGFR-2)	4ASD	Sorafenib	X = −18.033 Y = 3.988 Z = −15.404	*Homo sapiens*	Transferase	[59]
Stromelysin-1 (MMP-3)	1B3D	Hydroxamate-based inhibitor	X = −24.339 Y = 27.503 Z = −2.403	*Homo sapiens*	Hydrolase/hydrolase inhibitor	[60]
Tyrosinase-related protein 1 (TYRP1)	5M8M	Kojic acid	X = −31.100 Y = −6.213 Z = −25.609	*Homo sapiens*	Oxidoreductase	[43]

## Data Availability

The original contributions presented in this study are included in the article. Further inquiries can be directed to the corresponding author.

## Appendix A

**Table A1:** Binding energies (kcal/mol) obtained by molecular docking of *Chelidonium majus* L. compounds with the main proteins involved in wound healing.

Compounds	COX-2	iNOS	VEGF	STROMELYSIN-1	Tirosinase
**1**	−6.0	−5.9	−5.2	−6.4	−6.1
**2**	−5.9	−5.9	−4.8	−6.1	−5.2
**3**	−5.8	−6.7	−5.3	−6.1	−5.5
**4**	−5.7	−6.4	−5.9	−6.1	−5.5
**5**	−6.0	−6.0	−5.1	−6.3	−5.4
**6**	−7.1	−8.7	−7.3	−7.8	−6.1
**7**	−7.0	−6.4	−6.0	−6.1	−6.2
**8**	−7.4	−7.8	−7.8	−8.8	−7.2
**9**	−6.8	−6.8	−6.0	−6.6	−6.2
**10**	−6.3	−5.8	−5.3	−5.6	−5.5
**11**	−6.5	−6.5	−6.4	−6.5	−5.8
**12**	−6.9	−6.2	−6.3	−5.1	−5.8
**13**	−7.8	−9.5	−8.4	−9.1	−8.1
**14**	−6.7	−7.0	−6.5	−5.2	−5.8
**15**	−6.8	−7.3	−6.3	−6.6	−5.9
**16**	−8.4	−8.4	−8.2	−6.5	−7.0
**17**	−8.5	−8.9	−8.0	−8.8	−6.7
**18**	−8.1	−8.3	−6.9	−6.7	−6.1
**19**	−9.1	−9.6	−6.9	−9.5	−7.3
**20**	−9.3	−9.0	−7.8	−9.1	−6.6
**21**	−8.6	−8.5	−8.0	−9.3	−6.8
**22**	−4.5	−9.3	−9.4	−7.8	−6.9
**23**	−9.5	−9.6	−6.5	−8.4	−6.5
**24**	−8.5	−8.5	−7.7	−7.5	−6.2
**25**	−9.3	−10.1	−7.5	−9.6	−6.6
**26**	−6.0	−10.5	−7.7	−7.6	−7.8
**27**	−6.1	−9.0	−8.0	−7.8	−7.7
**28**	−2.6	−10.4	−9.2	−10.2	−6.2
**29**	−3.9	−10.8	−7.1	−9.1	−7.5
**30**	−4.8	−10.1	−8.0	−7.4	−6.9
**31**	−8.4	−8.9	−8.2	−7.2	−6.6
**32**	−9.9	−9.7	−7.0	−9.5	−6.3
**33**	−8.1	−9.6	−7.9	−7.4	−6.2
**34**	−8.4	−9.9	−7.1	−8.1	−5.7
**35**	−8.5	−9.9	−8.4	−9.6	−7.5
**36**	−6.9	−10.2	−8.4	−7.6	−6.1
**37**	−4.7	−11.2	−8.6	−8.3	−7.0
**38**	−7.4	−10.7	−7.6	−7.1	−6.4
**39**	−4.6	−11.4	−8.4	−8.8	−7.7
**40**	−6.9	−9.0	−8.1	−7.5	−6.7
**41**	−6.7	−9.2	−7.8	−8.1	−6.2
**42**	−5.1	−10.6	−7.3	−7.1	−5.8
**43**	−7.9	−10.2	−7.2	−7.6	−6.7
**44**	−9.7	−9.5	−7.3	−7.3	−6.4
**45**	−9.9	−9.5	−8.2	−7.6	−6.5
**46**	−6.9	−9.0	−7.0	−7.3	−6.4
**47**	−7.3	−7.2	−6.5	−7.5	−6.5
**48**	−7.2	−7.2	−6.1	−7.6	−6.5
**49**	−6.8	−7.0	−5.1	−7.0	−5.6
**50**	−5.7	−6.9	−5.5	−7.1	−6.2
**51**	−8.3	−8.7	−8.2	−8.2	−6.5
**52**	−8.8	−8.5	−6.9	−6.7	−6.9
**53**	−5.5	−5.7	−4.7	−5.2	−5.2
**54**	−5.6	−6.2	−4.7	−5.4	−5.0
**55**	−5.7	−5.9	−5.6	−5.8	−5.6
**56**	−5.7	−6.4	−5.9	−6.1	−5.5
**57**	−5.8	−6.7	−4.8	−5.5	−5.3
**58**	−4.0	−10.5	−8.6	−6.7	−6.0
**59**	−3.9	−9.1	−7.2	−7.3	−5.7
**60**	−1.5	−9.0	−6.5	−7.0	−5.4
**61**	−4.8	−9.9	−8.3	−7.8	−6.1
**62**	−3.3	−9.5	−7.4	−6.8	−5.9
**63**	−2.3	−10.2	−8.7	−7.9	−7.3
**64**	−5.3	−10.5	−8.4	−8.4	−6.0
**65**	−2.9	−9.5	−7.3	−7.3	−7.0
**66**	−0.4	−9.6	−7.5	−8.1	−6.4
**69**	−4.2	−7.4	−5.8	−6.4	−5.2
**70**	−4.2	−8.8	−6.7	−6.5	−5.2
**71**	−4.2	−10.3	−7.2	−7.5	−6.0
**72**	−6.8	−10.5	−8.0	−6.9	−6.6
**73**	−5.2	−10.0	−8.2	−8.1	−6.4
**74**	−6.4	−5.7	−5.9	−6.7	−6.4
**75**	−5.0	−9.1	−6.5	−6.3	−6.0
**76**	−3.0	−9.8	−6.5	−6.6	−6.0
**77**	−5.5	−10.1	−8.2	−8.4	−7.4
**78**	−3.1	−11.6	−8.6	−8.7	−6.8
**79**	−1.4	−8.1	−7.0	−7.4	−5.4
**80**	−7.8	−8.1	−6.4	−6.7	−6.4
**81**	−4.0	−9.2	−6.5	−7.2	−5.3
**82**	−8.9	−9.0	−6.6	−8.2	−6.9
**83**	−7.3	−7.9	−5.7	−7.7	−5.9
**84**	−6.6	−7.2	−5.8	−6.1	−6.5
**85**	−6.0	−7.8	−5.6	−7.5	−6.1
Control	−10.2	−5.6	−9.3	−6.2	−5.8

Binding affinity values are expressed in kcal/mol. Green-shaded cells indicate docking scores superior to the control ligand (representing stronger binding affinity through more negative thermodynamic values), while red-shaded cells denote values inferior to the control. The orange row highlights the control ligand’s results as a reference for comparison across all columns.

**Table A2:** Root-Mean-Square Deviation (RMSD) results for redocking experiments to validate the docking methodology.

Target	PDB ID	Co-Crystalizated Ligand (Control)	Resolution (Å)	RMSD (Å) *
COX-2	5IKR	Mefenamic Acid	2.34	0.958
iNOS	4NOS	Ethylisothiourea	2.25	0.936
VEGFR-2	4ASD	Sorafenib	2.03	0.742
MMP-3	1B3D	Hydroxamate-based inhibitor	1.90	1.499
TYRP1	5M8M	Kojic acid	2.35	2.115

* RMSD: Root-Mean-Square Deviation. A measure of the structural difference between the predicted docking pose and the experimental crystallographic position. It is used here to assess the reliability of the docking methodology.
